# Two Novel [^68^Ga]Ga-Labeled Radiotracers Based on Metabolically Stable [Sar^11^]RM26 Antagonistic Peptide for Diagnostic Positron Emission Tomography Imaging of GRPR-Positive Prostate Cancer

**DOI:** 10.1021/acsomega.4c01348

**Published:** 2024-04-10

**Authors:** Panagiotis Kanellopoulos, Ekaterina Bezverkhniaia, Ayman Abouzayed, Ulrika Rosenström, Vladimir Tolmachev, Anna Orlova

**Affiliations:** † Department of Medicinal Chemistry, Uppsala University, Uppsala 751 23, Sweden; ‡ Department of Immunology, Genetics and Pathology, Uppsala University, Uppsala 752 37, Sweden; § Science for Life Laboratory, Uppsala University, Uppsala 752 37, Sweden

## Abstract

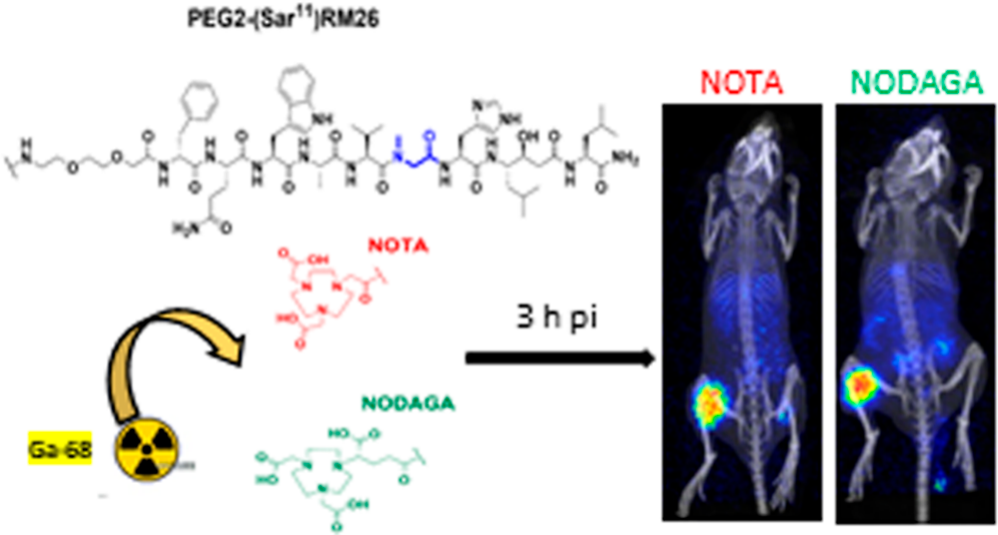

Gastrin releasing peptide receptor (GRPR) is overexpressed in prostate cancer (PC-3) and can be used for diagnostic purposes. We herein present the design and preclinical evaluation of two novel NOTA/NODAGA-containing peptides suitable for labeling with the positron emission tomography (PET) radionuclide Ga-68. These analogs are based on the previously reported GRPR-antagonist DOTAGA-PEG2-[Sar^11^]RM26, developed for targeted radiotheraostic applications. Both NOTA-PEG2-[Sar^11^]RM26 and NODAGA-PEG2-[Sar^11^]RM26 were successfully labeled with Ga-68 and evaluated in vitro and in vivo using PC-3 cell models. Both, [^68^Ga]Ga-NOTA-PEG2-[Sar^11^]RM26 and [^68^Ga]Ga-NODAGA-PEG2-[Sar^11^]RM26 displayed high metal-chelate stability in phosphate buffered saline and against the EDTA-challenge. The two [^68^Ga]Ga-labeled conjugates demonstrated highly GRPR-mediated uptake in vitro and in vivo and exhibited a slow internalization over time, typical for radioantagonistis. The [^nat^Ga]Ga-loaded peptides displayed affinity in the low nanomole range for GRPR in competition binding experiments. The new radiotracers demonstrated biodistribution profiles suitable for diagnostic imaging shortly after administration with fast background clearance. Their high tumor uptake (13 ± 1 and 15 ± 3% IA/g for NOTA and NODAGA conjugates, respectively) and high tumor-to-blood ratios (60 ± 10 and 220 ± 70, respectively) 3 h pi renders them promising PET tracers for use in patients. Tumor-to-normal organ ratios were higher for [^68^Ga]Ga-NODAGA-PEG2-[Sar^11^]RM26 than for the NOTA-containing counterpart. The performance of the two radiopeptides was further supported with the PET/CT images. In conclusion, [^68^Ga]Ga-NODAGA-PEG2-[Sar^11^]RM26 is a promising PET imaging tracer for visualization of GRPR-expressing lesions with high imaging contrast shortly after administration.

## Introduction

Prostate cancer (PC-3) (PCa) is among the most common malignancies in males, worldwide.^1^ Even though it is a rather common disease, its diagnosis still has limitations, mainly due to the availability of and the inherent shortcomings of tools used. Prostate specific antigen levels in blood, biopsies, and ultrasound imaging are some of the most used techniques for detection of PC-3 in patients. Still those approaches have their own detection-limits and their artifacts, lowering their diagnostic accuracy.^2−5^ An emerging field trying to cover this gap and provide the clinicians with more options into their repertoire is nuclear medicine. A field dedicated to the development of radiopharmaceuticals aiming for diagnosis with the use of single photon emission tomography (SPECT) or positron emission tomography (PET), and/or therapy with the deployment of radiotoxic payloads to tumor sites.^6−8^

One of the major aims of radiopharmaceutical chemistry is the development of radioligands designed to specifically deliver the radioactive isotope, suitable for diagnostic imaging or radiotherapy, on tumor sites, sparing any healthy tissue. Thus, high and specific binding to their biomolecular targets and a stable in vivo complexation of the radiometal utilized are of the outmost importance. Two of the most commonly used biomolecules serving as targets for radiopharmaceutical development for Pca are prostate specific membrane antigen (PSMA) and gastrin releasing peptide receptor (GRPR).^9−11^ Over the past years, PSMA was recognized as clinically relevant biomolecular-target in management of PCa, with the first FDA approved radiopharmaceuticals designed for diagnosis and therapy being PSMA-targeting radioligands (LOCAMETZ, Pluvicto for imaging and therapy, respectively).^12−14^ It is known that PSMA expression in PCa is strongly associated with later stages of the disease, while in the early stages of the malignancy, its expression is rather low.^12−14^

On the other hand, GRPR is proven to have higher expression in the early stages of PCa, which is even retained on metastatic sites.^15−17^ The inverse correlation between the two biomolecules indicates that not only both are valid as targets for PCa therapy and/or diagnosis (theranostics) but also they are complementary to each other.^18,19^

Over the past years, many attempts were made to design radiopeptides targeting GRPR. Although the preclinical results were encouraging, the pharmacological responses associated with the administration of radioagonists during the initial clinical trials led to the abandonment of this approach. The shift of the field from agonists to antagonistis for various receptors gave a new impetus on the quest for GRPR-targeting radioligands. The development of GRPR-antagonists, such as RM2 and RM26, gave new templates for the further development of novel GRPR-driven radiopharmaceuticals. After the introduction of these peptides, many attempts have been made to further improve their biological profile with the aim to increase activity uptake in tumors, while optimizing the overall pharmacokinetics.^20,21^

**Figure 1:**
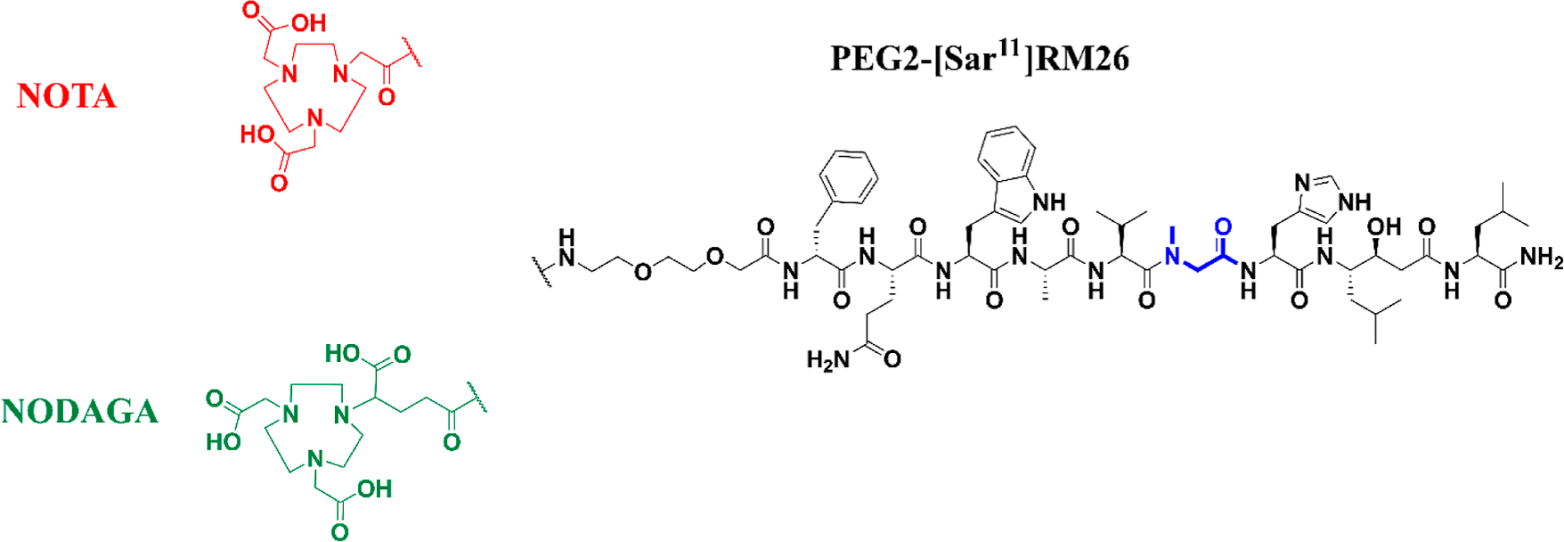
Chemical structures of NOTA-PEG2-[Sar^11^]RM26 and NODAGA-PEG2-[Sar^11^]RM26.

One such attempt resulted in the design of the AU-RM26-M1 (DOTAGA-PEG2-[Sar^11^]RM26).^22^ This peptide, based on RM26 sequence (d-Phe-Gln-Trp-Ala-Val-Gly-His-Sta-Leu-NH_2_,^23^ is bearing a DOTAGA chelator, excellent for labeling with radiometals suitable both for imaging (e.g., In-111 for SPECT) and therapy (e.g., Lu-177). The introduction of a sarcosine (Sar) at position 11 on the peptide sequence (d-Phe-Gln-Trp-Ala-Val-Sar-His-Sta-Leu-NH_2_) greatly increased its in vivo stability, thus, increasing tumor activity uptake. On top of that, the hydrophilic linker (PEG2) between chelator and GRPR-targeting moiety ensured a rapid clearance from the background though the urinary system.^24^ Intrigued by the rapid clearance from the healthy tissues with a rather high tumor uptake, we hypothesized that exchange of the DOTAGA chelator (2-(4,7,10-tris(carboxymethyl)-1,4,7,10-tetraazacyclododecan-1-yl)pentanedioic acid) with a chelator more suitable for Ga-68 labeling should result in the development of a PET tracer able to visualize GRPR-expressing lesions with high contrast shortly after administration. For that reason, we have chosen to use NOTA (1,4,7-triazacyclononane-1,4,7-triacetic acid) and NODAGA (2-(4,7-bis(carboxymethyl)-1,4,7-triazonan-1-yl)pentanedioic acid) chelators, due to the high stability of their complexes with Ga-68.^25^ Thus, two new peptides (NOTA-PEG2-[Sar^11^]RM26 and NODAGA-PEG2-[Sar^11^]RM26) (Figure 1) were designed, labeled with Ga-68, and tested preclinically both in vitro and in vivo in PC-3 cells and the PC-3 xenograft murine model.

## Materials and Methods

### Chemicals and Reagents

The peptides NOTA-PEG2-[Sar^11^]RM26, NODAGA-PEG2-[Sar11]RM26 and NOTA-PEG2-RM26 were synthesized by Pepmic Co., Ltd. (Suzhou, China). The GRPR-positive PC-3 cell line (PC-3) was purchased from the American Type Culture Collection (Manassas, VA, USA). Cell growth medium Roswell Park Memorial Institute (RPMI) 1640, fetal bovine serum (FBS), penicillin–streptomycin, and 6-well and 12-well plates were purchased from VWR International (Radnor, PA, USA). Trypsin −0.25% EDTA solution was obtained from Biochrom AG (Berlin, Germany). [^125^I]I-Tyr^4^-Bombesin and the Wizard2TM gamma counter were from PerkinElmer (Waltham, MA, USA). All other reagents were of chemical grade. Ga-68 was acquired by elution of an IGG100 Ge-68/Ga-68 generator provided by Eckert & Ziegler (Berlin, Germany).

### Ga-68/nat Labeling and Complex Stability Studies

Peptides were dissolved in MQ water to a final concentration of 1 mM and 50 μL aliquots were stored at −20 °C. For labeling with Ga-68, 2–5 μL of either NOTA-PEG2-[Sar^11^]RM26 or NODAGA-PEG2-[Sar^11^]RM26, were mixed with 250 μL sodium acetate (1.25 M, pH 3.6) and 250 μL of generator eluate (83 ± 8 MBq, measured after labeling). After thorough mixing, the reaction mixture was left to incubate at 85 °C for 15 min. After the incubation period, the labeling solution was left to cool down. Yields and purity were measured with instant thin layer chromatography (iTLC) and reverse phase high performance liquid chromatography (HPLC), respectively. For loading the conjugates with ^nat^Ga, 30 μL of each of the peptides was mixed with 30 μL [^nat^Ga]Cl_3_ (3 mM), 170 μL HCl (0.1 M), 250 μL sodium acetate (1.25 M, pH 3.6) and left to incubate for 20 min at 80 °C.

For iTLC-SG analysis, glass microfiber chromatography paper impregnated with silica gel by Agilent Technologies (Santa Clara, CA, USA) was used in combination with citric acid (0.2 M), as the mobile phase. Analysis of the iTLC stripes was performed by using Cycle Plus (PerkinElmer, Hägersten, Sweden).

The HPLC system used comprised a LaPrep Sigma HPLC LP1100 pump (Hitachi High-Tech Corporation, Hitachinaka, Ibaraki, Japan), a 40D LWL UV-detector with a 4 μL flow cell (Knauer, Berlin, Germany), a Flow scan radioactivity detector (Bioscan) with an FC-3300 NaI/PMT radioactivity probe (Eckert & Ziegler, Berlin, Germany), and a manual simple injector 7725i (Rheodyne) fitted with a 20 μL loop (IDEX Health & Science, LLC, CA, USA). The column used was a Luna C18 column (5 μm, 100 Å, 150 × 4.6 mm from Phenomenex, Værløse, Denmark). The gradient elution implemented, started at 95% A/5% B and reached 40% A/60% B over 20 min, where A is 0.1% v/v aqueous trifluoroacetic acid (TFA) and B is 0.1% v/v TFA in acetonitrile (MeCN).

The complex stability was tested in phosphate buffered saline (PBS) and in a 1000-fold excess of ethylenediaminetetraacetic acid (EDTA). In short, 10 μL of the labeling solution (corresponding to 40 pmol of peptide) dissolved in either 20 μL PBS or in 15 μL PBS and 5 μL Na_2_-EDTA (20 mM). After thorough mixing, the samples were left to incubate for 1 h at room temperature. After incubation, the release of Ga-68 was evaluated using iTLC. The statistical difference between PBS and EDTA was evaluated using an unpaired two-tailed TTest.

Mass spectra for [^nat^Ga]Ga-NOTA-PEG2-RM26, [^nat^Ga]Ga-NOTA-PEG2-[Sar^11^]RM26, and [^nat^Ga]Ga-NODAGA-PEG2-[Sar^11^]RM26 were acquired using Waters LCT Premier time-of-flight (TOF) mass spectrometer using electrospray ionization (ESI) and are included in the Supporting Information in Figures S1–S3, respectively.

### Cell Cultures

PC-3 cells were maintained in RPMI medium, containing glutamine, and supplemented with 10% w/w of FBS and 1% penicillin (10,000 U/mL)—streptomycin solution (10,000 μg/mL). Cultures were kept in a humidified environment at 37 °C and 5% CO2 using a Sanyo MCO-19AIC incubator (SANYO Electric Co., Ltd., Osaka City, Osaka, Japan). Cells were subcultured when in approximately 95% confluency, using Trypsin −0.25% EDTA solution.

### Cellular Uptake and in Vitro Specificity

For evaluation of the cellular uptake of the radioconjugates, 1 × 10^6^ PC-3 cells/well were seeded the day before, in six-well plates, and left to grow overnight. The day of the experiment, the cells were washed with 1 mL of PBS in room temperature and then a solution containing complete medium and the radiopeptide under evaluation (0.25 nM) and left to incubate at 37 °C for predetermined time points. After the supernatant was discarded, the cells were wash with 1 mL ice cold PBS, and then they were incubated twice with 600 μL acid wash (glycine 0.2 M, NaCl 0.15 M, urea 4 M, pH 2) for 5 min over ice and the supernatants were collected (membrane bound fraction—MB). After washing with 1 mL PBS, which was discarded, the cells were detached using 600 μL NaOH 1 M, twice (internalized fraction—Int).

For cell specificity, cells were seeded, as mentioned above. After washing with PBS, the cells were left to incubate in the presence of 1 mL of complete medium containing the radiopeptide under evaluation, and in the case of “block” samples, NOTA-PEG2-RM26 (25 nM) was also included as GRPR-blocking agent. Cells were left to incubate at 37 °C for 1 h. After discarding the supernatant, cells were detached using trypsin—EDTA solution, and the suspension was collected in 5 mL RIA tubes.

Measurement of the radioactivity of the samples was performed using the Wizard2TM gamma counter (PerkinElmer, Waltham, MA, USA). For statistical analysis two-way ANOVA with Tuckey’s post hoc analysis was performed using GraphPad Prism v7 for Windows (GraphPad Software, Boston, Massachusetts USA).

### Competition Binding Experiments

In a 12-well plate, 5 × 10^5^ PC-3 cells/well were seeded and left to proliferate overnight. The following day, after removal of the supernatant, cells were washed with cold PBS (4 °C) and 350 μL of PBS supplemented with 1% w/v bovine serum albumin (BSA) was introduced to the cells—binding buffer (BB). Next, 50 μL of a solution of the compound under investigation in BB was added, with concentrations ranging 0–5000 nM. Finally, 100 μL of [^125^I]I-Tyr4-BBN (100,000 cpm, 24.6 fmol) in BB were introduced and the cells were left to incubate at 4 °C for 5 h. After the supernatant was removed and the cells were washed with cold PBS, cells were collected using a trypsin—EDTA solution. The radioactivity content of the samples was measured by using a gamma counter. Data analysis and curve fitting were done with GraphPad Prism 7 using the nonlinear regression model. For statistical analysis one-way ANOVA with Tuckey’s posthoc analysis was used.

### Biodistribution and Imaging Studies

In vivo studies were conducted in accordance with European guidelines on laboratory animal protection and the Declaration of Helsinki. For the biodistribution and PET/CT experiments BALB/C nu/nu mice were used and the protocols were approved by the ethics committee for animal research in Uppsala (Sweden); permit number 00473/21.

After acclimatization, animals were subcutaneously injected on the right hind leg with a suspension of freshly harvested PC-3 cells (7 × 10^6^ per animal) in PBS. Four weeks after, well palpable solid tumors were developed and the animals were randomly assigned in groups of four.

Biodistribution studies of [^68^Ga]Ga-NOTA-PEG2-[Sar^11^]RM26 and [^68^Ga]Ga-NODAGA-PEG2-[Sar^11^]RM26 were performed in 1 and 3 h post injection (pi). Animals (19 ± 2 g at the day of the experiment) were injected through the tail vein with 100 μL solution of radiopeptide under evaluation (40 pmol, 100 kBq–1 h pi, 40 pmol, 700 kBq–3 h pi) in PBS containing 1% w/v BSA. Another group of animals received a bolus injection with the radioconjugate (40 pmol, 300 kBq) in addition of 4 nmol of NOTA-PEG2-RM26–1 h pi Block group. At specified time points, animals were euthanized, tissues/organs of interest and tumors were collected, weighted, and the radioactivity content of the samples was measured.

Statistical analysis for the biodistribution data was performed with GraphPad Prism 7, employing a two-way ANOVA test with Tuckey’s posthoc analysis.

For imaging studies, two PC-3 xenograft bearing mice, one per radioligand, were injected with 100 pmol of peptide, corresponding to 3 MBq of radioactivity, and whole body static PET/CT images were taken at 1 and 3 h pi. Nano PET 3T (PET/MRI) and nanoScan (SPECT/CT) apparatus were from Mediso Medical Imaging Systems (Budapest, Hungary). Reconstruction of the PET scans was conducted using Nucline nanoScan 3.04.014.0000 software. CT data were reconstructed using Filter Back Projection in Nucline 2.03 Software (Mediso Medical Imaging Systems Ltd., Budapest, Hungary). PET and CT files were fused using Nucline 2.03 Software and are presented as maximum intensity projections on the RGB color scale.

## Results

### Labeling of NOTA-PEG2-[Sar^11^]RM26 and NODAGA-PEG2-[Sar^11^]RM26 with Ga-68 and Complex Stability Test

**Figure 2:**
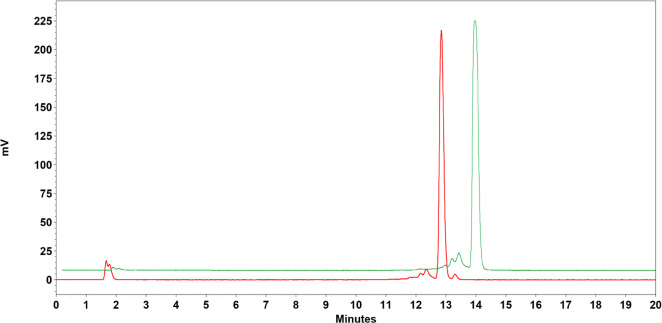
Chromatograms of [^68^Ga]Ga-NOTA-PEG2-[Sar^11^]RM26 (red) and [^68^Ga]Ga-NODAGA-PEG2-[Sar^11^]RM26 (green) after labeling (no decay correction done).

Radiochemical yields for the labeling of NOTA-PEG2-[Sar^11^]RM26 and NODAGA-PEG2-[Sar^11^]RM26 with Ga-68 were over 95%, as determined by iTLC analysis (Table 1). During incubations with PBS or in the presence of 1000-fold excess of EDTA, little-to-no release of free Ga-68 from the chelators was observed (Table 1). The radiochemical purity of both radioligands was in all cases >95%, as determined by radio-HPLC analysis (Figure 2).

**Table 1:** Radiochemical Characterization of [^68^Ga]Ga-NOTA-PEG2-[Sar^11^]RM26 and [^68^Ga]Ga-NODAGA-PEG2-[Sar^11^]RM26^a^

compound	RCY (*n*) (iTLC)	PBS (*n*)^b^	EDTA (*n*)
[^68^Ga]Ga-NOTA-PEG2-[Sar^11^]RM26	95.9 ± 0.8% (6)	97 ± 3% (3)^b^	93.9 ± 0.1% (3)^b^
[^68^Ga]Ga-NODAGA-PEG2-[Sar^11^]RM26	98 ± 2 (9)	97 ± 1% (3)^b^	96.9 ± 0.7% (3)^b^

a *n*: Number of Repetitions.

b No statistical difference was observed between the groups.

### Cellular Uptake and in Vitro GRPR-Specificity Test

**Figure 3:**
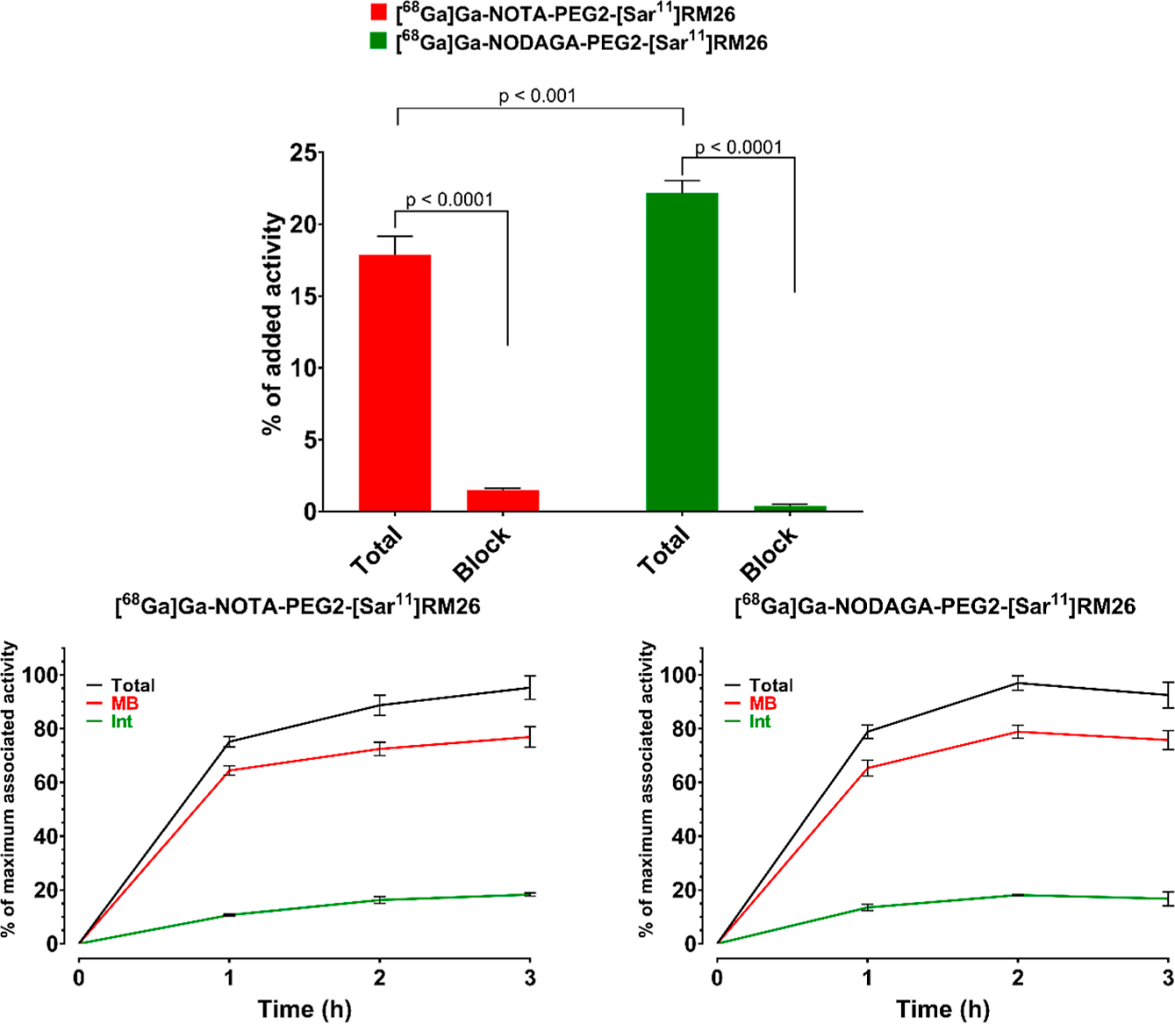
(A) Specificity test for [^68^Ga]Ga-NOTA-PEG2-[Sar^11^]RM26 and [^68^Ga]Ga-NODAGA-PEG2-[Sar^11^]RM26 in PC-3 cells. (B) Cell-association profile of [^68^Ga]Ga-NOTA-PEG2-[Sar^11^]RM26 in PC-3 cells, over time, (C) cell-association profile of [^68^Ga]Ga-NODAGA-PEG2-[Sar^11^]RM26 in PC-3 cells, over time. MB: membrane bound, Int: internalized.

Cellular uptake of [^68^Ga]Ga-NOTA-PEG2-[Sar^11^]RM26 and [^68^Ga]Ga-NODAGA-PEG2-[Sar^11^]RM26 were highly GRPR-mediated as can be seen in the in vitro specificity test (Figure 3A). Cell associated activity was significantly lower if cells were pretreated with GRPR-binding ligand before addition of radiolabeled peptides (*p* < 0.0001 in both cases).

Both radioconjugates had quite a high uptake in PC-3 cells (Figure 3B,C). The bulk of the cell associated activity remained on the cell-surface in all instances and the internalized fraction slowly increased overtime. Among the two tested peptides, [^68^Ga]Ga-NODAGA-PEG2-[Sar^11^]RM26 had higher cellular uptake (22.9 ± 0.1% of added activity being cell-associated after 1 h of incubation) than [^68^Ga]Ga-NOTA-PEG2-[Sar^11^]RM26 (18 ± 1%) (experiments were performed in parallel using cells seeded from the same suspension, *p* < 0.001).

### Competition Binding Assay

**Figure 4:**
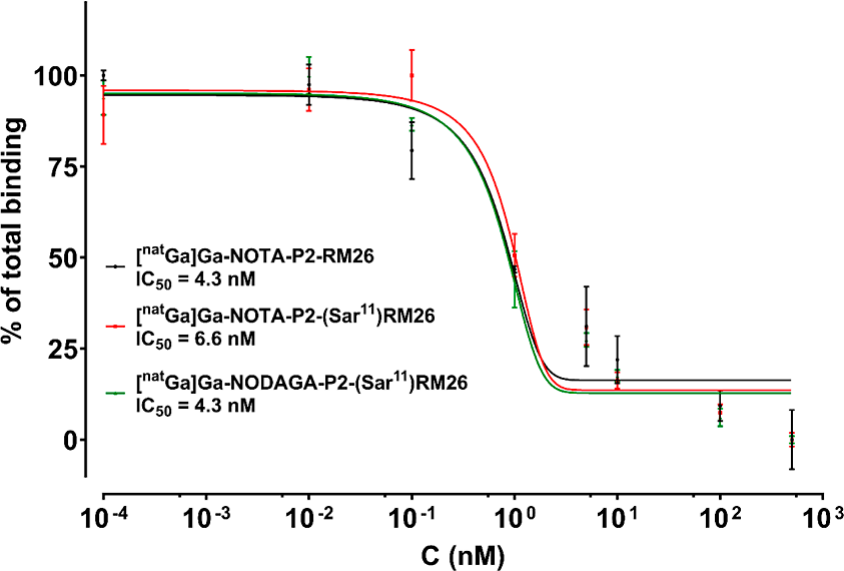
Competition binding curves of [^nat^Ga]Ga-NOTA-PEG2-RM26 (black), [^nat^Ga]Ga-NOTA-PEG2-[Sar^11^]RM26 (red), and [^nat^Ga]Ga-NODAGA-PEG2-[Sar^11^]RM26 (green). All three compounds were tested again with [^125^I]I-Tyr^4^-bombesin on live PC-3 cells. Experiments were performed in triplicates.

Both NOTA- and NODAGA-PEG2-[Sar^11^]RM26 were loaded with natural gallium and their IC_50_ values were determined against [^125^I]I-Tyr4-bombesin on live PC-3 cells. As an internal control, [^nat^Ga]Ga-NOTA-PEG2-RM26^26^ was also used. As it was proven by electron spray mass spectroscopy for all three analogs, gallium was in complex with the peptide, without free-chelator remaining (Figures S1–S3). All three metalated compounds displayed IC_50_ values in the single digit nanomolar range (Figure 4). Assays were performed in parallel using the cells from the same passage and the same solution of displacement-ligand. There was no statistically significant difference between the obtained IC_50_ values (*p* > 0.05 for all comparisons).

### Biodistribution and Imaging Experiments

**Figure 5:**
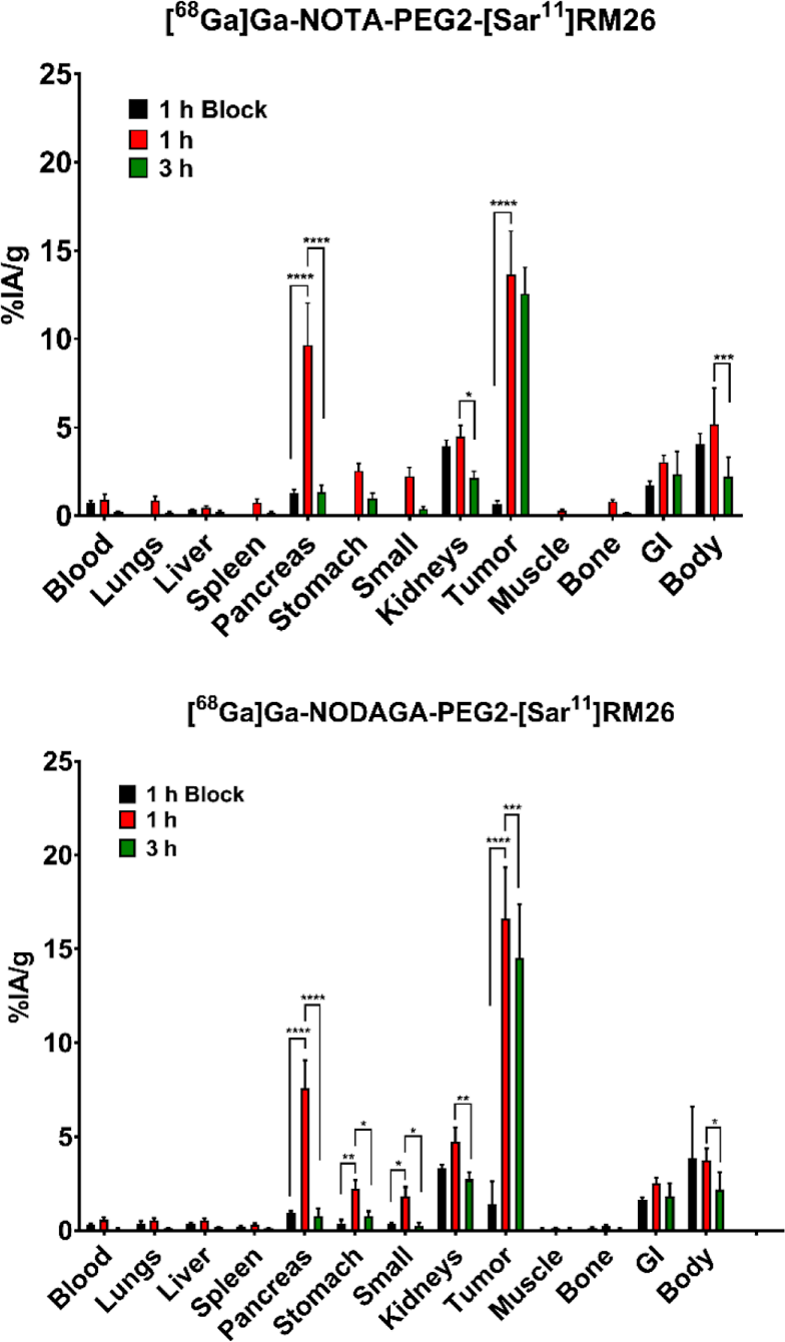
Graphical representation of biodistribution data for [^68^Ga]Ga-NOTA-PEG2-[Sar^11^]RM26 and [^68^Ga]Ga-NODAGA-PEG2-[Sar^11^]RM26. Small: small intestines, GI: gastrointestinal tract (excluding stomach and part of small intestines); body is measured after excision of the major organs presented. Values for body and GI are given as % IA. *: *p* < 0.05; **: *p* < 0.01; ***: *p* < 0.001; ****: *p* < 0.0001.

The biodistribution profiles for [^68^Ga]Ga-NOTA-PEG2-[Sar^11^]RM26 and [^68^Ga]Ga-NODAGA-PEG2-[Sar^11^]RM26 in PC-3 tumor bearing mice at 1 and 3 h pi are presented on Figure 5. Both [^68^Ga]Ga-labeled analogs had a very fast background clearance with values for blood activity concentration being below 1% IA/g at 1 h pi, which further dropped at 3 h pi. Excretion of radiopeptides was predominantly via the urinary system, as indicated by the low activity uptake in liver (about 0.5% IA/g) and somewhat elevated uptake in kidneys (below 5% IA/g). Values for activity uptake in kidneys were 3-fold lower than activity uptake in tumors already at 1 h pi in both instances, and decreased significantly at 3 h pi (*p* < 0.05 for NOTA and *p* < 0.01 for NODAGA-containing ligand).

The activity uptake in the pancreas, an organ with high GRPR expression, was elevated at 1 h pi, still being 2-fold lower than activity uptake in tumors, and significantly decreased at 3 h pi. Other GRPR-rich organs, such as stomach and small intestines, displayed low activity uptake even at 1 h pi. Organs and tissues without the expression of GRPR demonstrated very low activity uptake.

The activity uptake for tumors was the highest among the tested organs and tissues. Tumor activity uptake at 1 h pi reached 14 ± 3% IA/g for [^68^Ga]Ga-NOTA-PEG2-[Sar^11^]RM26 and 17 ± 3% IA/g for [^68^Ga]Ga-NODAGA-PEG2-[Sar^11^]RM26. The uptake was at persistently high levels between 1 and 3 h pi, with the respective values for the two radiotracers being 13 ± 2% IA/g and 15 ± 3% IA/g (*p* > 0.5 & *p* < 0.001) at 3 h pi.

Significant decrease for activity uptake in pancreas and tumors was observed when radiolabeled conjugates were coinjected with excess of nonlabelled high affinity GRPR-binding agent (*p* < 0.0001 in all cases). This confirmed a highly GRPR-specific activity uptake for both radioconjugates. Biodistribution data are summarized in Figure 5 and Tables S1 and S2.

**Figure 6:**
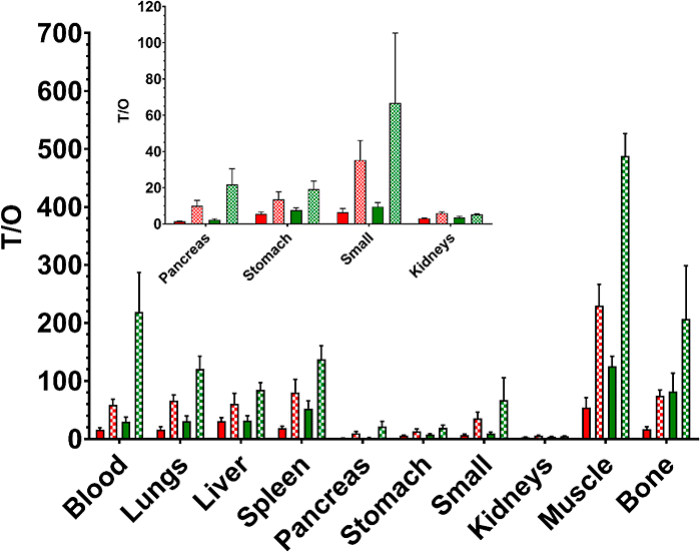
Tumor to organ ratios (T/O) for [^68^Ga]Ga-NOTA-PEG2-[Sar^11^]RM26 and [^68^Ga]Ga-NODAGA-PEG2-[Sar^11^]RM26. Red: [^68^Ga]Ga-NOTA-PEG2-[Sar^11^]RM26 1 h pi; red-checkered: [^68^Ga]Ga-NOTA-PEG2-[Sar^11^]RM26 3 h pi; green: [^68^Ga]Ga-NODAGA-PEG2-[Sar^11^]RM26 1 h pi; green-checkered: [^68^Ga]Ga-NODAGA-PEG2-[Sar^11^]RM26 3 h pi. Small: small intestines.

[^68^Ga]Ga-NODAGA-PEG2-[Sar^11^]RM26 having faster background clearance in combination with higher activity uptake in tumors displayed higher tumor-to-organ ratios than the NOTA-bearing conjugate (Figure 6, Table S3). That fact was even more evident at 3 h pi, and the mean ratios for tumor-to-nontumor were at least 1.5-fold higher for [^68^Ga]Ga-NODAGA-PEG2-[Sar^11^]RM26 than the ones for [^68^Ga]Ga-NOTA-PEG2-[Sar^11^]RM26.

**Figure 7:**
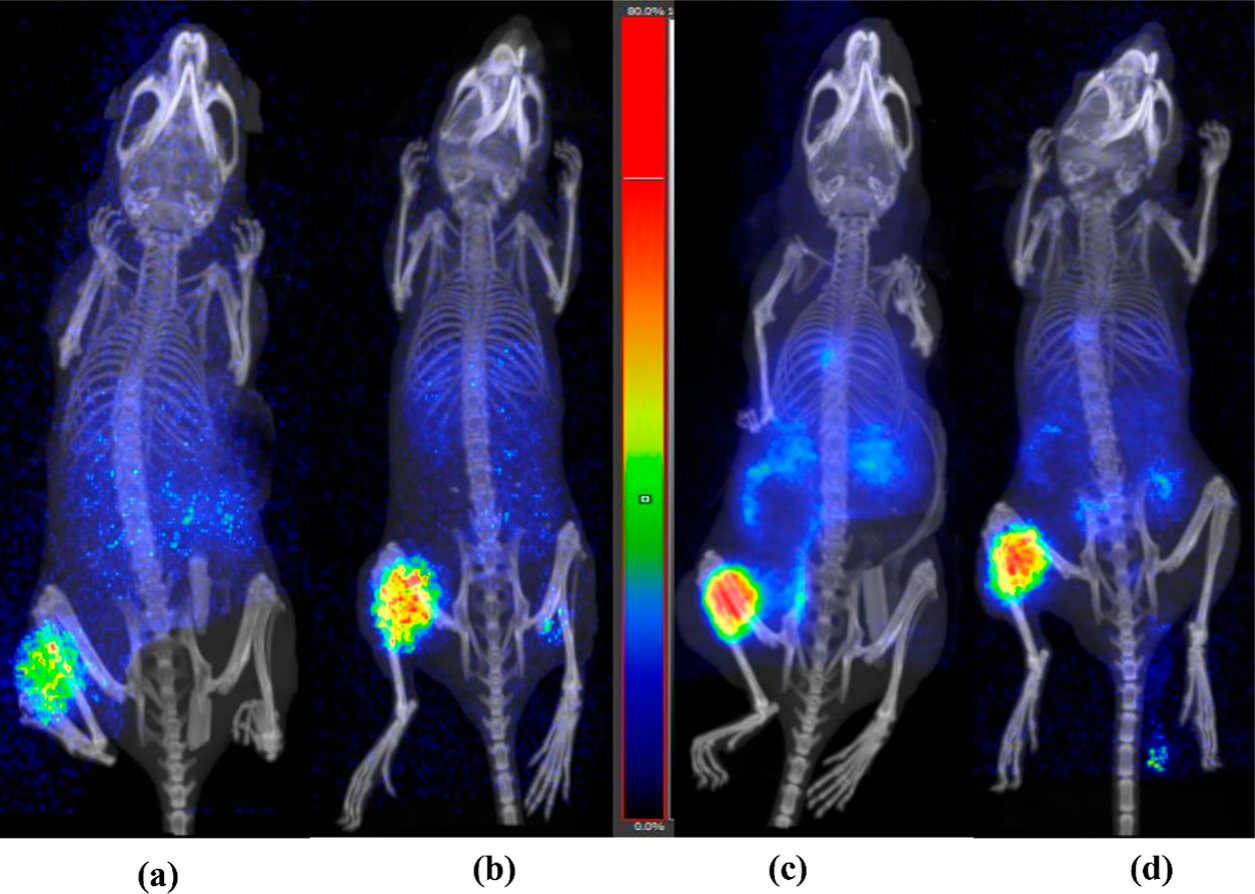
PET/CT images of: (a,b) [^68^Ga]Ga-NOTA-PEG2-[Sar^11^]RM26 (1 and 3 h pi, respectively) (c,d) [^68^Ga]Ga-NODAGA-PEG2-[Sar^11^]RM26 (1 and 3 h pi, respectively), in PC-3 xenograft bearing mice. Images’ scale was adjusted to 80% of max. Urinary bladder was masked in all animals.

Following the biodistribution studies, static PET/CT images for the two radiopeptides were acquired at 1 and 3 h pi. As it is evident from the images (Figure 7), both radiotracers had an excellent performance in vivo, with [^68^Ga]Ga-NODAGA-PEG2-[Sar^11^]RM26 having an edge over [^68^Ga]Ga-NOTA-PEG2-[Sar^11^]RM26, due to more clear background. Images were in good agreement with the ex vivo biodistribution data.

## Discussion

Radiolabeled PSMA inhibitors have been recently approved for PCa management, labeled with diagnostic radionuclides Ga-68 and F-18 and with therapeutic radiometal Lu-177.^27,28^ This fact gave a great boost on the appeal of targeted radionuclide therapy and theranostics in general. The introduction of those radiopharmaceuticals provided new means for personalized treatment in the clinicians’ arsenal against PCa. Despite the huge success, the fact that PSMA expression level is strongly associated with later stages of PCa, hinders their efficacy in detection of lesions, both primary and metastatic, in the onset of the disease. More and more attention is drawn on development of GRPR-targeting radiopharmaceuticals, since GRPR is shown to be overexpressed in the majority of cases of PCa, especially in early stages, with retained expression both in primary and metastatic sites.^15−17^

Being part of the effort to provide new GRPR-targeting radiopharmaceuticals, our group has recently reported the development of a novel In-111 labeled radioconjugate suitable for SPECT diagnostic imaging based on the RM26 motif. The DOTAGA chelator was used in this conjugate, allowing further labeling with therapeutic radiometals, such as Lu-177 or Ac-225. As demonstrated [^111^In]In-DOTAGA-PEG2-[Sar^11^]RM26 had a very fast background clearance with decently high tumor retention overtime in mice bearing PC-3 xenografts.^22^ Given these promising results in diagnostic potential, the question raised if the same peptide sequence could be used effectively in the design of novel PET tracer(s), utilizing NOTA/NODAGA-chelator in combination with Ga-68, due to the higher sensitivity provided by PET. To answer that question, two novel radiotracers were designed, NOTA-PEG2-[Sar^11^]RM26 and NODAGA-PEG2-[Sar^11^]RM26.

Both radiopeptides were successfully labeled with Ga-68 and their metal-chelate complexes’ stability was very high as it was proven by the low activity uptake in bones in vivo (<0.9% IA/g for both conjugates). Cellular uptake was highly GRPR-mediated with a typical for radioantagonistist uptake profile, with the bulk of the cell-associated activity remaining on the membrane and a slow internalization fraction over time.

Competition binding experiments for the two metalated peptides, against the well-established [^125^I]I-Tyr^4^-bombesin, showed IC_50_ values in single digit nanomolar range, almost identical with the one of [^nat^Ga]Ga-NOTA-PEG2-RM26, which served as a reference due to its high affinity for GRPR.^24,26^ It is interesting to note that half inhibition concentration for [^nat^Ga]Ga-NOTA-PEG2-RM26 was slightly, but significantly better for that for [^nat^Ga]Ga-NODAGA-PEG2-RM26 when compared head-to-head.^24^ This was attributed to the positive charge of the Ga-NOTA complex. However, in the present study [^nat^Ga]Ga-NODAGA-PEG2-[Sar^11^]RM26 and [^nat^Ga]Ga-NOTA-PEG2-[Sar^11^]RM26 did not show any difference, since both had IC_50_ values almost identical to [^nat^Ga]Ga-NOTA-PEG2-RM26. This could reflect the uncertainty in the peptide concentration determination in the working solution. Nevertheless, both new peptides demonstrated a high binding affinity to GRPR.

When the biodistribution profiles of the two radiopeptides were tested in mice bearing GRPR-expressing xenografts of PCa origin, both conjugates had high pancreatic and tumor uptakes at 1 h pi. This could be expected due to their high affinity for GRPR. In both cases, tumor values were at least 2-fold higher than those for pancreas and much higher than for other anatomically relevant for PCa-imaging organs/tissues, i.e., muscle, bones, intestine. Both radiopeptides had a rapid clearance from the background, including from the GRPR-expressing organs such as small intestines, stomach, and pancreas, while tumor values were retained more or less on the same level at 3 h pi. Tumor activity uptake at 3 h pi was 6-fold higher than in kidneys for [^68^Ga]Ga-NOTA-PEG2-[Sar^11^]RM26 and 5.3-fold higher for [^68^Ga]Ga-NODAGA-PEG2-[Sar^11^]RM26. Both radiotracers displayed high in vivo specificity, as demonstrated by the highly significant decrease in activity uptake in tumors and other GRPR-expressing organs after the coadministration of an excess of NOTA-PEG2-RM26.

It is quite interesting to compare the biodistribution results for the two new conjugates with their counterparts without the Gly^11^/Sar^11^ substitution, i.e., [^68^Ga]Ga-NOTA-PEG2-RM26 and [^68^Ga]Ga-NODAGA-PEG2-RM26, previously reported.^24^ Despite the similar amounts of peptide and activity injected, the biodistribution data for [^68^Ga]Ga-NOTA-PEG2-RM26 and [^68^Ga]Ga-NODAGA-PEG2-RM26, in PC-3 bearing mice, are reported only for 2 h pi.^24^ Under these conditions, we are going to focus on a comparison with the data for 3 h pi. In comparison between the two sets of compounds, NOTA or NODAGA-coupled peptides, the Sar^11^-substituded ones show more or less the same uptake in the majority of organ/tissues of interest. On the other hand, there is a pronounced difference for the tumor uptake, due to higher in vivo stability of the radiopeptides after the introduction of Sar at position 11. In detail, the values for tumor uptake of [^68^Ga]Ga-NOTA-PEG2-RM26 were 5 ± 1% IA/g at 2 h pi, where, for [^68^Ga]Ga-NOTA-PEG2-[Sar^11^]RM26 are 13 ± 1% IA/g at 3 h pi. For the second set, the respective values were [^68^Ga]Ga-NODAGA-PEG2-RM26–3.9 ± 0.7% IA/g (2 h pi); [^68^Ga]Ga-NODAGA-PEG2-[Sar^11^]RM26–15 ± 3% IA/g (3 h pi). Thus, increase of in vivo stability of the new conjugates by substitution of Gly^11^ by Sar^11^, led to 2.4-fold and 3.8-fold increase of the median activity uptake for the tumors for the NOTA- and NODAGA-coupled radioconjugates,^24^ respectively.

Based on the aforementioned promising results, one could say that the approach taken to translate [^111^In]In-DOTAGA-PEG2-[Sar^11^]RM26 into [^68^Ga]Ga-labeled PET tracer(s) was rather successful. Both compounds performed excellent, with [^68^Ga]Ga-NODAGA-PEG2-[Sar^11^]RM26 taking the lead in terms tumor targeting in vivo and overall tumor-to-organ ratios. The fact that the NODAGA-coupled radiopeptide performed better than the NOTA-coupled radiopeptide is rather intriguing. A positive charge is anticipated for NOTA in complex with Ga-68, while the Ga-NODAGA complex should be neutral. From the literature it is expected that the positive charge at the N-terminus should be favored by the GRPR.^24,29^

On this point, a comparison with other [^68^Ga]Ga-labeled radioligands would be of interest. Especially in comparison with GRPR-targeting radioconjugates tested in clinical trials, like: [^68^Ga]Ga-SB3 (DOTA-*p*-aminomethylaniline-diglycolic acid-DPhe-Gln-Trp-Ala-Val-Gly-His-Leu-NHEt),^30,31^ [^68^Ga]Ga-RM2 (DOTA-Pip-DPhe-Gln-Trp-Ala-Val-Gly-His-Sta-Leu-NH_2_),^17,32,33^ and [^68^Ga]Ga-NOTA-PEG3-RM26 (NOTA-PEG3-d-Phe-Gln-Trp-Ala-Val-Gly-His-Sta-Leu-NH_2_).^19,24,34^ One should be careful with those comparisons, though. In these studies, different strains of mice, different batches of PC-3 cells, and even different injected activities and masses were used; thus, a head-on comparison is not that straightforward and it needs to be made with caution.

In preclinical setting, [^68^Ga]Ga-NOTA-PEG2-[Sar^11^]RM26 and [^68^Ga]Ga-NODAGA-PEG2-[Sar^11^]RM26 demonstrated a tumor/pancreas ratio over 1 already 1 h pi, similar to [^68^Ga]Ga-NOTA-PEG3-RM26, while [^68^Ga]Ga-SB3, [^68^Ga]Ga-NODAGA-MJ9 and [^68^Ga]Ga-RM2 had negative contrast with pancreas (1 h pi activity uptake in pancreas was 4-, 3- and 2-fold higher than in tumors, respectively). In comparison with [^68^Ga]Ga-SB3, both [^68^Ga]Ga-NOTA-PEG2-[Sar^11^]RM26 and [^68^Ga]Ga-NODAGA-PEG2-[Sar^11^]RM26 had lower tumor uptake but also had lower uptake for kidneys and pancreas, thus resulting in a clearer background. When compared with [^68^Ga]Ga-RM2 or [^68^Ga]Ga-NODAGA-MJ9, tumor and kidney values were on par.^29,30,32^

In agreement with their preclinical data, SUV values for the pancreas for [^68^Ga]Ga-SB3 and [^68^Ga]Ga-RM2 were around 40, while for [^68^Ga]Ga-PEG3-RM26, this value was 2-fold lower. All three clinically tested compounds demonstrated tumor/kidney ratios about 3 as early as 1 h pi in murine models and a low to moderate activity accumulation was confirmed clinically.^17,31,33^

Based on the comparison above, it is expected for [^68^Ga]Ga-NOTA-PEG2-[Sar^11^]RM26 and [^68^Ga]Ga-NODAGA-PEG2-[Sar^11^]RM26 to have very promising clinical performances, presumably even better than the radioligands mentioned above.

## Conclusions

In conclusion, both NOTA-PEG2-[Sar^11^]RM26 and NODAGA-PEG2-[Sar^11^]RM26 were successfully labeled with Ga-68 and evaluated both in vitro and in vivo. Based on their biodistribution profile, a very promising tumor-targeting capability was shown from both compounds, a bit better for [^68^Ga]Ga-NODAGA-PEG2-[Sar^11^]RM26, with very fast background clearance. Based on the above-mentioned information, both are valid candidates for clinical translation.

## Supporting Information Available

Biodistribution data for [^68^Ga]Ga-NOTA-PEG2-[Sar^11^]RM26, [^68^Ga]Ga-NODAGA-PEG2-[Sar^11^]RM26, and T/O ratios for [^68^Ga]Ga-NOTA-PEG2-[Sar^11^]RM26 and [^68^Ga]Ga-NODAGA-PEG2-[Sar^11^]RM26; and mass spectrum of [^nat^Ga]Ga-NOTA-PEG2-RM26, [^nat^Ga]Ga-NOTA-PEG2-[Sar^11^]RM26, and [^nat^Ga]Ga-NODAGA-PEG2-[Sar^11^]RM26 (PDF)

## Abbreviations

BSA: bovine serum albumin
CT: computed tomography
DOTAGA: 2-(4,7,10-tris(carboxymethyl)-1,4,7,10-tetraazacyclododecan-1-yl)pentanedioic acid
EDTA: ethylenediamine-tetraacetic acid
FBS: fetal bovine serum
FDA: Food and Drug Administration
GRPR: gastrin releasing peptide receptor
HPLC: reverse phase high performance liquid chromatography
IC_50_: inhibition concentration 50%
iTLC: instant thin layer chromatography
NODAGA: 2-(4,7-bis(carboxymethyl)-1,4,7-triazonan-1-yl)pentanedioic acid
NOTA: 1,4,7-triazacyclononane-1,4,7-triacetic acid
PBS: phosphate buffered saline
PCa: prostate cancer
PET: positron emission tomography
pi: post injection
PSA: prostate specific antigen
PSMA: prostate specific membrane antigen
RCY: radiochemical yield
SPECT: single photon emission computed tomography
% IA/g: percentage of injected activity per g of tissue
