# Supplementary material for: Two Novel [68Ga]Ga-Labeled Radiotracers Based on Metabolically Stable [Sar11]RM26 Antagonistic Peptide for Diagnostic Positron Emission Tomography Imaging of GRPR-Positive Prostate Cancer
Source: ACS Omega. 2024 Apr 10;9(16):18608–16. doi: 10.1021/acsomega.4c01348 (PMC11044165; doi:10.1021/acsomega.4c01348)

Two novel [^{68}Ga]Ga-labeled radiotracers based on metabolically stable [Sar^{11}]RM26 antagonistic peptide for diagnostic PET imaging of GRPR-positive prostate cancer

Panagiotis Kanellopoulos[1], Ekaterina Bezverkhniaia[1], Ayman Abouzayed[1], Ulrika Rosenström[1], Vladimir Tolmachev[2], Anna Orlova[1,3]*

1 Department of Medicinal Chemistry, Uppsala University, 751 23 Uppsala, Sweden; ekaterina.bezverkhniaia@ilk.uu.se (E.B.); panagiotis.kanellopoulos@ilk.uu.se (P.K.); ayman.abouzayed@ilk.uu.se (A.A.); ulrika.rosenstrom@ilk.uu.se (U.R.); anna.orlova@ilk.uu.se (A.O.)

2 Department of Immunology, Genetics and Pathology, Uppsala University, 752 37 Uppsala, Sweden; vladimir.tolmachev@igp.uu.se (V.T.)

3 Science for Life Laboratory, Uppsala University, 752 37 Uppsala, Sweden

*Correspondence: anna.orlova@ilk.uu.se

Supplementary data

Table S1. Biodistribution data for [^{68}Ga]Ga-NOTA-PEG2-[Sar^{11}]RM26 in PC-3 xenograft bearing mice. GI: gastrointestinal track; GI and Body are given in %IA.

	**1 h Block**	***p***	**1 h**	***p***	**3 h**
**Blood**	0.75 ± 0.09	>0.05	0.9 ± 0.3	>0.05	0.22 ± 0.05
**Lungs**	ND		0.9 ± 0.2	>0.05	0.19 ± 0.03
**Liver**	0.34 ± 0.01	>0.05	0.5 ± 0.1	>0.05	0.22 ± 0.08
**Spleen**	ND		0.7 ± 0.2	>0.05	0.17 ± 0.07
**Pancreas**	1.3 ± 0.2	<0.0001	10 ± 2	<0.0001	1.3 ± 0.4
**Stomach**	ND		2.5 ± 0.4	>0.05	1 ± 0.3
**Small Intestines**	ND		2.2 ± 0.5	>0.05	0.4 ± 0.1
**Kidneys**	3.9 ± 0.4	<0.01	4.5 ± 0.6	<0.01	2 ± 0.4
**Tumor**	0.7 ± 0.2	<0.0001	14 ± 2	>0.05	13 ± 1
**Muscle**	ND		0.27 ± 0.08	>0.05	0.06 ± 0.01
**Bone**	ND		0.81 ± 0.08	>0.05	0.17 ± 0.03
**GI**	1.7 ± 0.2	>0.05	3 ± 0.4	>0.05	2 ± 1
**Body**	4.1 ± 0.6	>0.05	5 ± 0.2	<0.001	2 ± 1

ND: no data

Table S2. Biodistribution data for [^{68}Ga]Ga-NODAGA-PEG2-[Sar^{11}]RM26in PC-3 xenograft bearing mice. GI: gastrointestinal track; GI and Body are given in %IA.

	**1 h Block**	***p***	**1 h**	***p***	**3 h**
**Blood**	0.30 ± 0.06	>0.05	0.6 ± 0.1	>0.05	0.07 ± 0.03
**Lungs**	0.4 ± 0.1	>0.05	0.6 ± 0.1	>0.05	0.12 ± 0.02
**Liver**	0.38 ± 0.02	>0.05	0.5 ± 0.1	>0.05	0.17 ± 0.03
**Spleen**	0.21 ± 0.04	>0.05	0.33 ± 0.07	>0.05	0.11 ± 0.02
**Pancreas**	0.9 ± 0.1	<0.0001	8 ± 1	<0.0001	0.8 ± 0.4
**Stomach**	0.4 ± 0.2	<0.01	2.2 ± 0.5	<0.05	0.8 ± 0.2
**Small Intestines**	0.35 ± 0.06	<0.05	1.8 ± 0.5	<0.05	0.3 ± 0.1
**Kidneys**	3.3 ± 0.2	>0.05	4.7 ± 0.8	<0.01	2.7 ± 0.4
**Tumor**	1 ± 1	<0.0001	17 ± 3	<0.001	15 ± 3
**Muscle**	0.09 ± 0.04	>0.05	0.13 ± 0.02	>0.05	0.07 ± 0.08
**Bone**	0.14 ± 0.03	>0.05	0.23 ± 0.09	>0.05	0.07 ± 0.04
**GI**	1.6 ± 0.2	>0.05	2.5 ± 0.3	>0.05	1.8 ± 0.7
**Body**	4 ± 3	>0.05	3.8 ± 0.6	<0.05	2.2 ± 0.9

Table S3 Tumor to organ ratios for [^{68}Ga]Ga-NOTA-PEG2-[Sar^{11}]RM26 and .[^{68}Ga]Ga-NODAGA-PEG2-[Sar^{11}]RM26.

T/O	[^{68}Ga]Ga-NOTA-PEG2-[Sar^{11}]RM26		[^{68}Ga]Ga-NODAGA-PEG2-[Sar^{11}]RM26	
	**1 h**	**3 h**	**1 h**	**3 h**
**Blood**	16 ± 3	59 ± 10	30 ± 8	219 ± 68
**Lungs**	16 ± 5	66 ± 10	31 ± 8	120 ± 22
**Liver**	31 ± 5	61 ± 18	32 ± 8	85 ± 12
**Spleen**	19 ± 3	80 ± 23	52 ± 14	137 ± 23
**Pancreas**	1.4 ± 0.2	10 ± 3	2.3 ± 0.5	22 ± 9
**Stomach**	5 ± 1	14 ± 4	8 ± 1	19 ± 5
**Small Intestines**	6 ± 2	35 ± 10	10 ± 2	67 ± 38
**Kidneys**	3 ± 0.3	6 ± 0.7	3.6 ± 0.7	5.3 ±0.4
**Muscle**	54 ± 17	230 ± 36	125 ± 17	488 ± 39
**Bone**	17 ± 4	74 ± 10	82 ± 31	207 ± 92

Figure S1. MS spectrum for [^{nat}Ga]Ga-NOTA-PEG2-RM26.

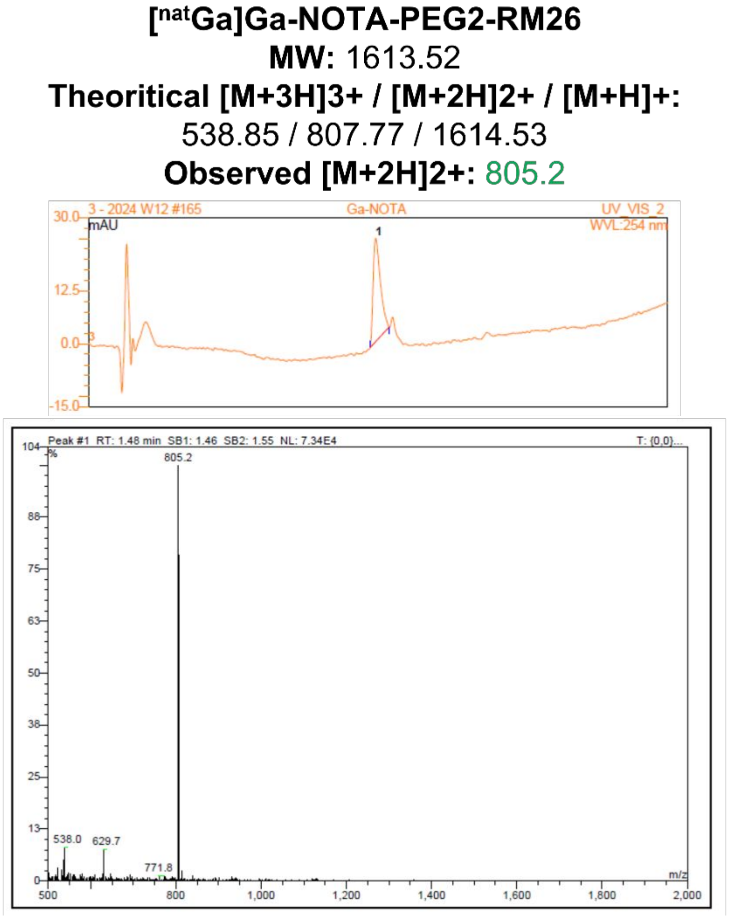

Figure S2. MS spectrum for [^{nat}Ga]Ga-NOTA-[Sar^{11}]PEG2-RM26.

[^{nat}Ga]Ga-NOTA-PEG2-[Sar^{11}]RM26
MW: 1557.80
Theoritical [M+3H]3+ / [M+2H]2+ / [M+H]+:
543.51 / 814.76 / 1628.51
Observed [M+2H]2+: 812.2

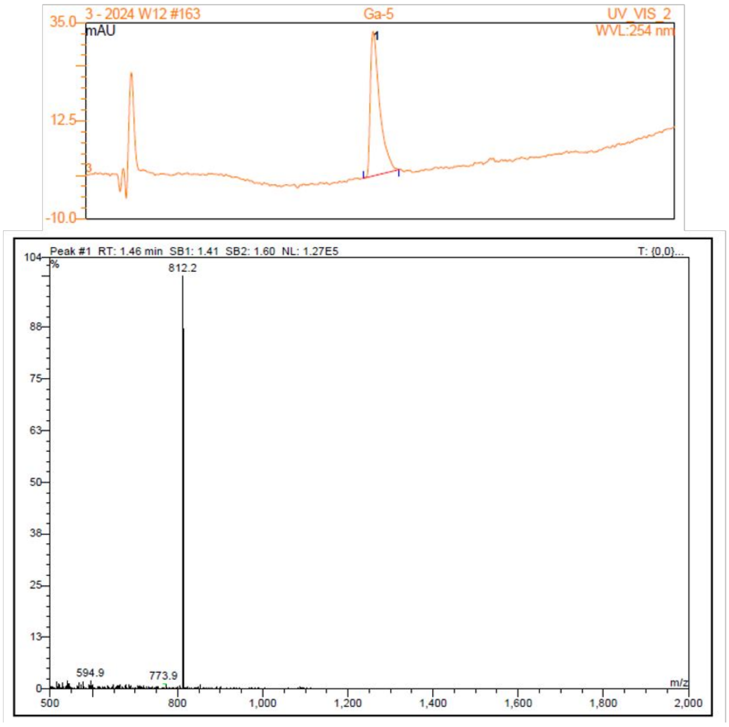

Figure S3. MS spectrum for [natGa]Ga-NODAGA-[Sar11]PEG2-RM26.

[natGa]Ga-NODAGA-PEG2-[Sar11]RM26

MW: 1699.6

Theoritical [M+3H]3+ / [M+2H]2+ / [M+H]+:

567.54 / 850.81 / 1700.60

Observed [M+2H]2+: 849.2

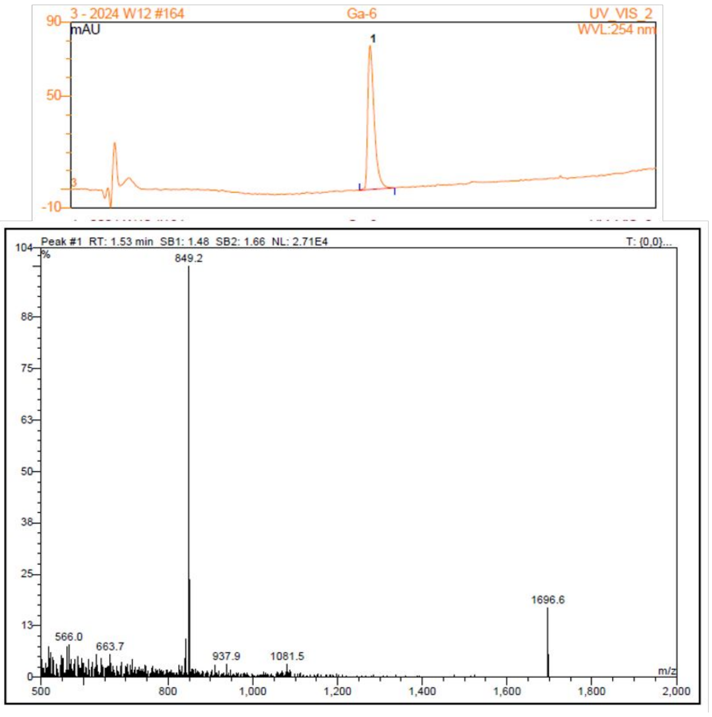

Supplement: Supplementary file 1 — ao4c01348_si_001.pdf [file ao4c01348_si_001.pdf]
